# A Case of Diffuse Sclerosing Osteomyelitis (DSO) Initially Diagnosed as Bacterial Mandibular Osteomyelitis and Successfully Treated With Bisphosphonate Administration (Ibandronate)

**DOI:** 10.7759/cureus.76210

**Published:** 2024-12-22

**Authors:** Kei-ichiro Miura, Misa Sumi, Masataka Umeda, Masahiro Umeda, Tomohiro Yamada

**Affiliations:** 1 Department of Oral and Maxillofacial Surgery, Nagasaki University Graduate School of Biomedical Sciences, Nagasaki University, Nagasaki, JPN; 2 Department of Radiology and Biomedical Informatics, Nagasaki University Graduate School of Biomedical Sciences, Nagasaki University, Nagasaki, JPN; 3 Department of Immunology and Rheumatology, Nagasaki University Graduate School of Biomedical Sciences, Nagasaki University, Nagasaki, JPN

**Keywords:** ct (computed tomography) imaging, diffuse sclerosing osteomyelitis, dso, intraosseous ibandronate, mandible osteomyelitis, mri imaging, oral and maxillofacial surgery, short term bisphosphonate

## Abstract

Diffuse sclerosing osteomyelitis (DSO) is a non-bacterial disease of the jawbone, characterized by intermittent pain, swelling, and a mixture of osteosclerosis and osteolysis on radiographs. Its etiology remains unclear, and a standard treatment, based on clear diagnostic criteria, has not been established. We present the case of a 48-year-old male patient, who was initially diagnosed with chronic mandibular osteomyelitis due to apical periodontitis in the right lower second premolar, and underwent antimicrobial medication and surgical therapy based on computed tomography (CT), magnetic resonance imaging (MRI), and bone scintigraphy. However, the bone resorption image, with a periosteal reaction, was enlarged on CT, and the area of accumulation was also increased on bone scintigraphy. The diagnosis was switched to DSO, bisphosphonate administration (ibandronate 1 mg/month) was initiated, and the pain improved one month after administration, with the bone resorption almost disappearing after six months. The patient has been doing well for four years and four months since the initial diagnosis. This case highlights the importance of prompt diagnosis and appropriate treatment of DSO, which differs from bacterial mandibular osteomyelitis. Bisphosphonate administration is an effective treatment that contributes to the long-term course of the disease.

## Introduction

Diffuse sclerosing osteomyelitis (DSO) is a nonbacterial disease characterized by intermittent pain and swelling of the jawbone, without abscess formation or pus discharge. DSO is refractory to antibacterial agent administration and surgical therapy [1], and radiologically shows a combination of osteosclerosis and osteolysis [2]. Therefore, early diagnosis and appropriate treatment are essential. However, because the etiology of DSO remains unclear, a standard treatment, based on clear diagnostic criteria, has not yet been established. In this report, we describe a case of DSO with a satisfactory response to bisphosphonate administration. This case was initially diagnosed as bacterial mandibular osteomyelitis caused by apical periodontitis, and no treatment effect was obtained by antibacterial administration and surgical treatment prior to bisphosphonate administration.

## Case presentation

**Figure 1:**
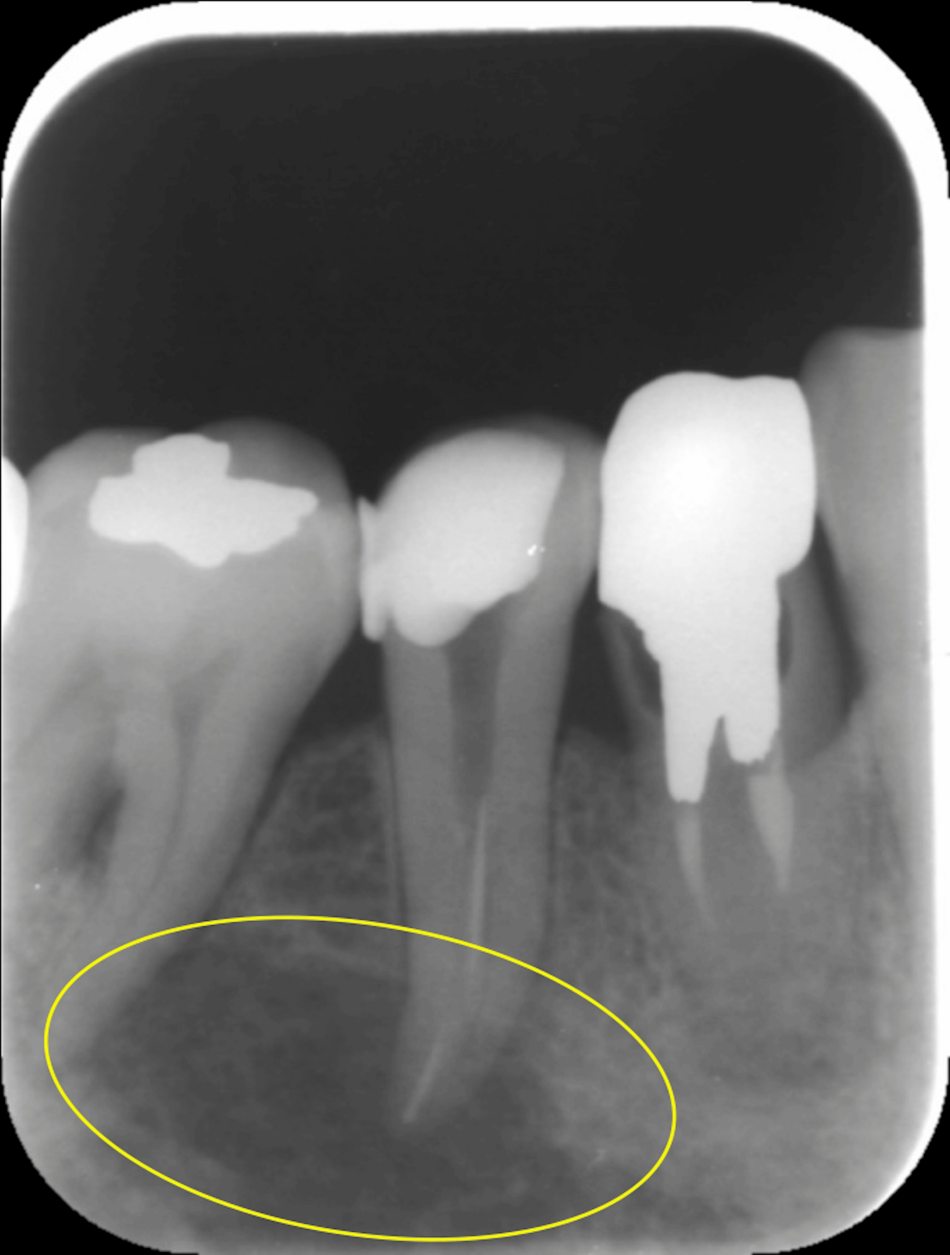
Periapical radiograph A radiolucent area is recognized, from the distal surface of the root of the right lower second premolar to the mesial surface of the mesial root of the right lower first molar.

**Figure 2:**
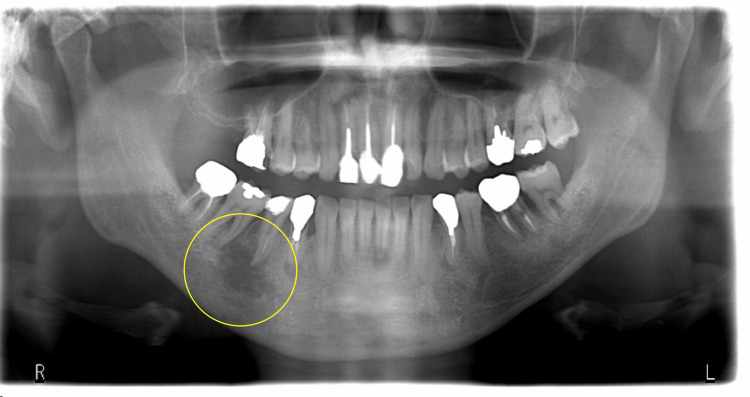
Panoramic X-ray A radiolucent area (yellow oval) is recognized, from the apical lesion of the right lower second premolar to that of the proximal root of the right lower first molar. The mandibular border is intact.

In March 2015, a 48-year-old male presented with pain in his lower right premolar and underwent root canal treatment at a local dentist. As the pain did not improve, the patient visited our department in April 2015. At the first visit, there were no specific extraoral findings. Intraoral findings showed tenderness in the gingiva of the lower right second premolar apical area, without swelling, redness, or pus discharge. A periapical radiograph revealed a fractured dental instrument in the tooth and a radiolucent area at the apical region (Figure 1), which also showed a radiolucent area at the apical region of the right lower second premolar in the panoramic X-ray (Figure 2).

**Figure 3:**
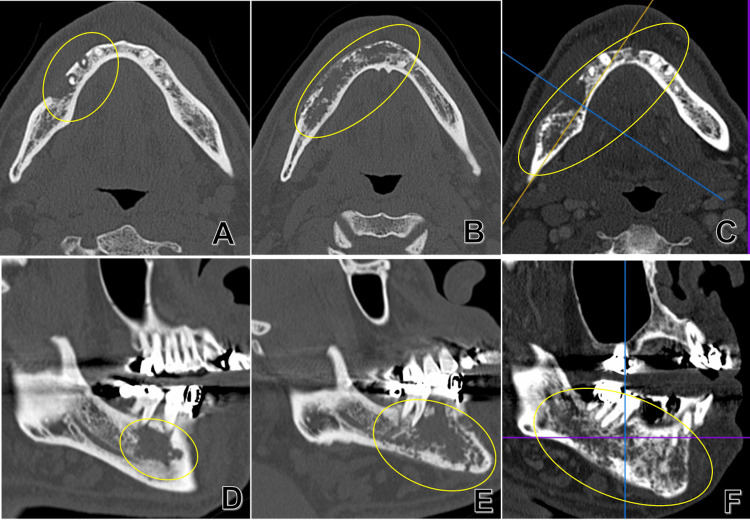
CT image CT images at initial examination (A, D), one month after tooth extraction of the right lower second premolar (B, E), and two months after extraction (C, F) show the enlargement of the bone resorption area (yellow oval) over time. CT, computed tomography

Computed tomography (CT) revealed a bone resorption area of approximately 2 cm, from the right lower second premolar to the mesial surface of the mesial root of the right lower first molar (Figures 3A, 3D). Based on these findings, we diagnosed apical periodontitis of the right lower second premolar and performed a lower right second premolar extraction, along with a biopsy of the granulation tissue of the apical lesion in April 2015. The histopathological diagnosis was granulation tissue. The structure of the radicular cyst was not recognized, and malignant findings were ruled out. Although the extraction socket epithelialized postoperatively, the area of pain expanded to the left side of the premolar area, and the administration of antibacterial agents and non-steroidal anti-inflammatory drugs (NSAIDs) was started in May 2015. Immediately after administration, the pain temporarily improved; however, it relapsed one month later, and the bone resorption area was enlarged on CT (Figures 3B, 3E). Two months after the tooth extraction, CT revealed diffuse bone destruction from the lower right molar region to the left incisal tooth area (Figures 3C, 3F).

**Figure 4:**
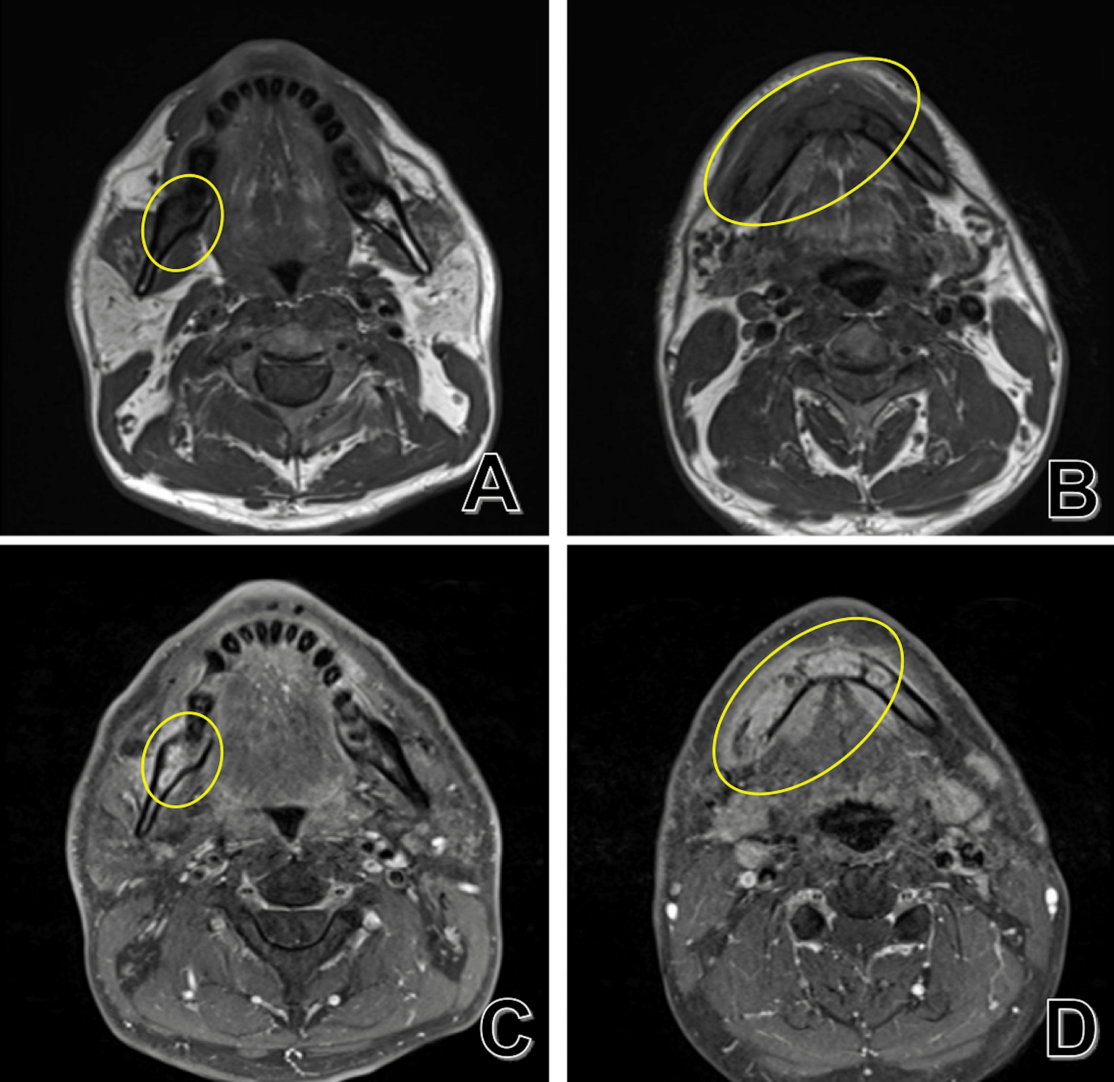
MRI T1-weighted images (A, B) and contrast-enhanced T1-weighted images (C, D) show an extensive low-signal area, recognized from the left lower first premolar to the right ramus (yellow oval). The inflammation area spreads to surrounding soft tissues, but no abscess formation is recognized. MRI, magnetic resonance imaging

**Figure 5:**
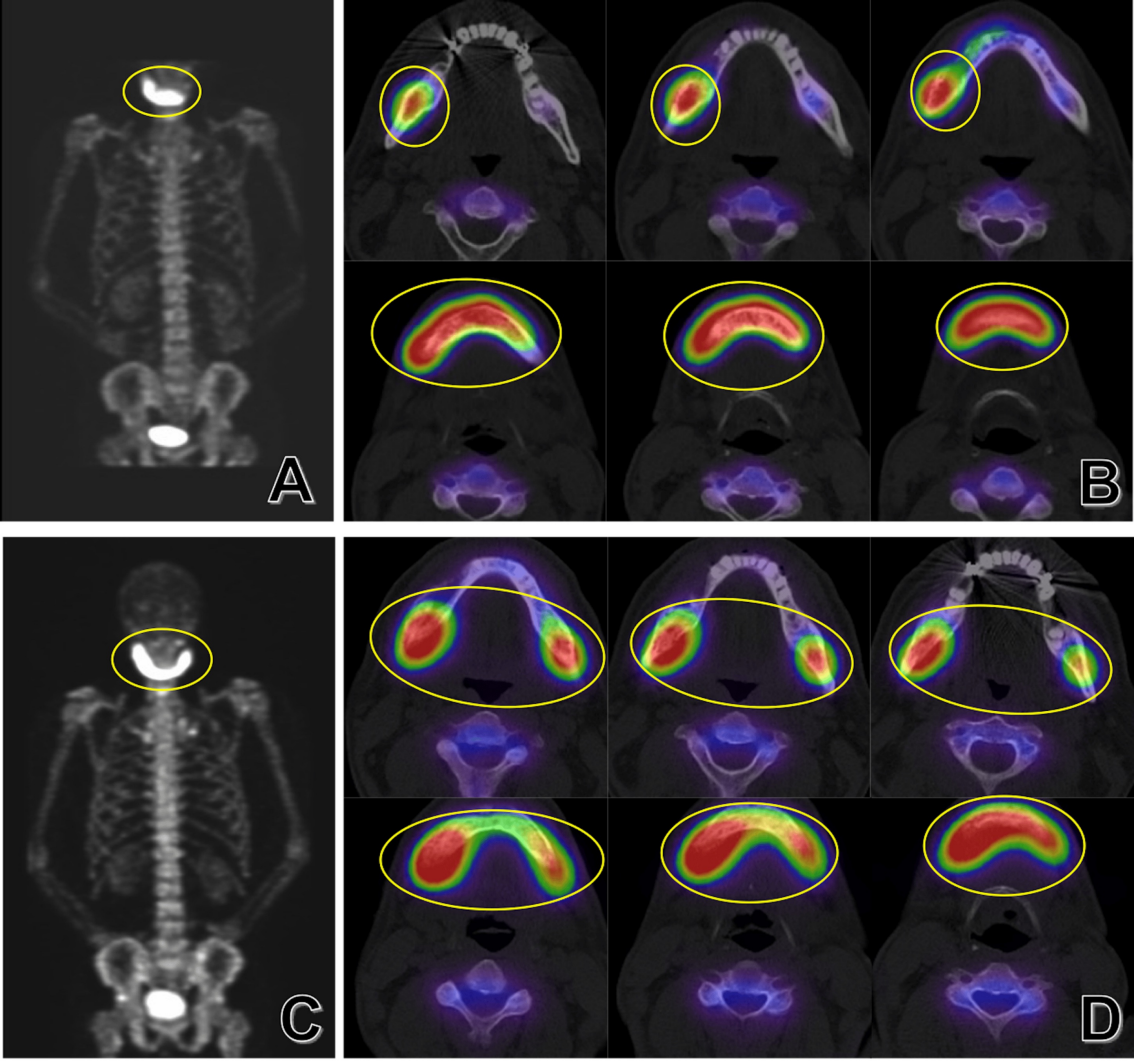
Bone scintigraphy image Preoperative (A, B) and six-month postoperative (C, D) images show the expansion of the accumulation area (yellow oval).

Therefore, magnetic resonance imaging (MRI) (Figures 4A-4D) and bone scintigraphy (Figures 5A-5B) were performed to further investigate the pathological condition, and mandibular osteomyelitis was diagnosed.

**Figure 6:**
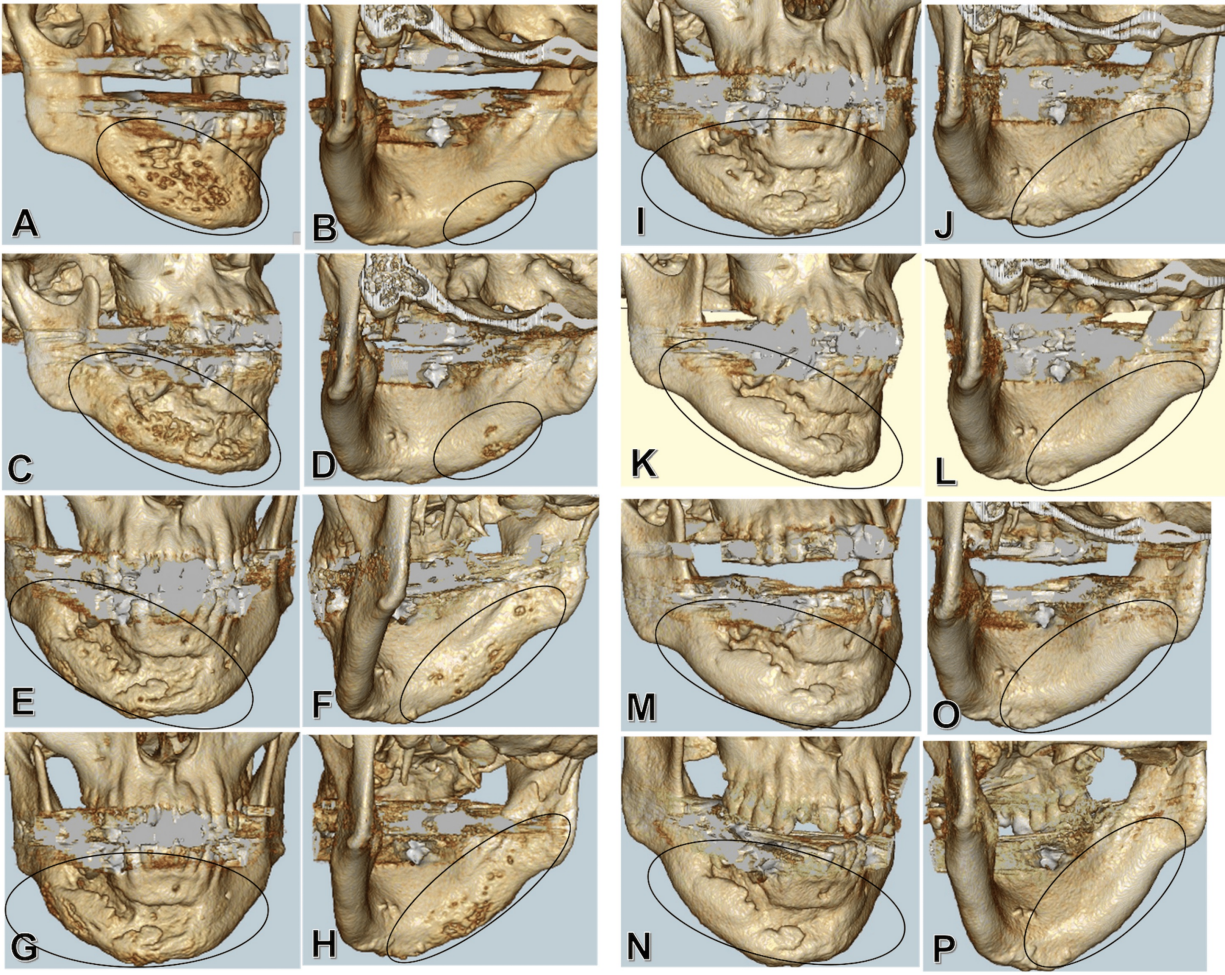
3D CT image 3D CT images before surgery (A, B), one month after surgery (C, D), three months after surgery (E, F), five months after surgery (G, H), two months after BP administration (I, J), six months after BP administration (K, L), one year after BP administration (M, O), and two years after BP administration (N, P) are shown. Postoperatively, the progression of cortical bone resorption in the right mandibular body is recognized. On the other hand, it has improved after BP administration. The area of interest is indicated as a black oval. CT, computed tomography; BP, bisphosphonate

Surgical decortication was performed in August 2015, and pathological specimens detected *Staphylococcus epidermidis*-MRCNS (methicillin-resistant coagulase-negative staphylococci) and *Streptococcus oralis*, which finally led to the diagnosis of bacterial mandibular osteomyelitis caused by the right lower second premolar apical periodontitis. Postoperative antibacterial medication was continued. However, since the bone resorption with periosteal reaction was enlarged on postoperative CT (Figures 6A-6H), and the area of accumulation had worsened on bone scintigraphy (Figures 5C-5D), we considered the necessity of a differential diagnosis of systemic diseases, such as SAPHO (synovitis, acne, pustulosis, hyperostosis, and osteitis) syndrome, and referred the patient to the Immunology and Rheumatology Department for consultation. In the additional evaluation, neither sacroiliitis on X-ray or MRI, nor symptoms suggesting peripheral arthritis or enthesitis, were observed, and the condition was diagnosed as DSO. Therefore, we concluded that the condition did not necessitate treatment with disease-modifying anti-rheumatic drugs (DMARDs) or biological agents. Based on reports demonstrating the effectiveness of intravenous ibandronate, a bisphosphonate, in managing bone resorption and pain associated with DSO [3,4], we initiated monthly intravenous injections of ibandronate at a dose of 1 mg in March 2016, and the pain improved one month after initiation.

In addition, the bone resorption area almost disappeared after six months. After ibandronate administration from March 2016 to February 2017, there was no recurrence of pain or bone resorption (Figures 6I-6P). Four years and four months have passed since the initial diagnosis, and the patient has remained uneventful.

## Discussion

Because the initial examination indicated bacterial mandibular osteomyelitis caused by apical periodontitis in the right lower second premolar, the patient was treated with antibacterial agents and surgical treatment. The standard treatment for chronic bacterial mandibular osteomyelitis aims to improve circulation and aerobic conditions, and surgical decortication is considered an appropriate radical treatment. However, the patient's pain did not improve postoperatively, and postoperative CT showed further bone resorption. As the administration of antibacterial agents was not effective, *S. epidermidis*-MRCNS and *S. oralis*, which were detected at the surgical site, were thought to be derived simply from contamination of oral flora, and the diagnosis of bacterial mandibular osteomyelitis needed to be switched. Therefore, we considered the possibility of SAPHO syndrome and performed a systemic examination, which did not reveal any lesions other than osteomyelitis of the jaw. We finally diagnosed this case as DSO because SAPHO syndrome has other systemic symptoms, such as acne, hyperostosis, synovitis, and pustulosis [5], and our case occurred in the mandible alone, which did not fulfill the diagnostic criteria for chronic recurrent multifocal osteomyelitis (CRMO) [6]. MRI findings are helpful for the diagnosis of DSO because DSO shows a low bone marrow signal on T1-weighted images [7] and the absence of abscess formation [8]. It has been proposed that increased osteoclast activity [7-9] and proinflammatory cytokines, such as IL-6, IL-20, and TNF-α, are involved in the pain and rapid bone resorption progression caused by DSO [9], and that these are suppressed by bisphosphonate administration, resulting in a therapeutic effect.

The current standard treatment for DSO with significant skeletal damage is intravenous bisphosphonate administration, and the effectiveness of ibandronate administration has been previously reported. A study of 11 patients found that single-shot infusions of ibandronate led to significant pain reduction within 48-72 hours, with most patients becoming free or almost free of complaints for months afterward [3]. A cross-sectional study of 15 DSO patients treated with 6 mg of ibandronate reported significantly improved quality of life, reduced pain, and decreased analgesic intake two weeks after infusion [10]. Another case report described a remarkable response to a single infusion of zoledronate (another bisphosphonate) in a patient with DSO, suggesting that bisphosphonates, as a class, may be effective [11]. The case report described satisfactory responses to ibandronate in three patients, with changes in bone markers, after alternative bisphosphonates had failed [10].

Recently, chronic non-bacterial osteitis (CNO) has been reported to share similarities with DSO. According to the 2024 expert consensus recommendations for the diagnosis and treatment of CNO in adults [12], intravenous bisphosphonate administration is recommended as a second-line treatment when NSAIDs are ineffective. Also, in these expert consensus recommendations [12], bone pain likely caused by osteitis needs to be assessed based on clinical symptoms and radiological measures of bone marrow edema. In this case report, the patient's pain persisted even after the extraction socket had healed. The origin of this pain was not thought to be bacterial mandibular osteomyelitis, but rather bone pain likely caused by osteitis due to DSO.

Since the diagnostic criteria and mechanisms of DSO are still unclear, there is a possibility that the initial treatment may be delayed, as seen in the present case. We need to continue gaining new knowledge for the rapid and accurate diagnosis of DSO and to differentiate it from bacterial mandibular osteomyelitis, SAPHO syndrome, and CRMO.

## Conclusions

DSO is a different condition from bacterial mandibular osteomyelitis and requires prompt diagnosis and appropriate treatment. Administration of bisphosphonate is an effective treatment that contributes to a good long-term course. In recent years, SAPHO syndrome, CRMO, and DSO have been defined as CNO, which is a term that describes a sterile bone inflammatory disease. Further research should be conducted in detail to continue providing good outcomes for patients.
